# High‐Performance Dielectric Elastomer Nanogenerator for Efficient Energy Harvesting and Sensing via Alternative Current Method

**DOI:** 10.1002/advs.202201098

**Published:** 2022-04-09

**Authors:** Zisheng Xu, Kunwei Bao, Kui Di, Haojie Chen, Jianbo Tan, Xinjun Xie, Yixin Shao, Jiancheng Cai, Shizhe Lin, Tinghai Cheng, Shiju E, Kang Liu, Zhong Lin Wang

**Affiliations:** ^1^ Key Laboratory of Urban Rail Transit Intelligent Operation and Maintenance Technology & Equipment of Zhejiang Province College of Engineering Zhejiang Normal University Jinhua 321004 P. R. China; ^2^ Jinhua Intelligent Manufacturing Research Institute Jinhua 321004 P. R. China; ^3^ Beijing Institute of Nanoenergy and Nanosystems Chinese Academy of Sciences Beijing 100083 P. R. China; ^4^ MOE Key Laboratory of Hydraulic Machinery Transients School of Power and Mechanical Engineering Wuhan University Wuhan 430072 P. R. China; ^5^ College of Nanoscience and Technology University of Chinese Academy of Sciences Beijing 100049 P. R. China; ^6^ School of Materials Science and Engineering Georgia Institute of Technology Atlanta GA 30332 USA

**Keywords:** charge density, dielectric elastomer generator, electret, energy harvesting, nanogenerator

## Abstract

Soft, low‐cost, high‐performance generators are highly desirable for harvesting ambient low frequency mechanical energy. Here, a dielectric elastomer nanogenerator (DENG) is reported, which consists of a dielectric elastomer capacitor, an electret electrostatic voltage source, and a charge pump circuit. Under biaxial stretching, DENG can convert tensile mechanical energy into electrical power without any external power supply. Different from traditional DEG with the charge outward transfer in direct current (DC), the DENG works based on shuttle movement of internal charges in an alternating current (AC). Through alternating current (AC) method, the charge density of the DENG can reach 26 mC m^−2^ per mechanical cycle, as well as energy density of up to 140 mJ g^−1^. Due to the all‐solid‐state structure without air gap, the DENG is capable of working stably under different ambient humidity (20 RH%–100 RH%). To demonstrate the applications, a water wave harvester based on the DENG is constructed. The integrated device powers a sensing communication module for self‐powered remote weather monitoring, showing the potential application in ocean wave energy harvesting.

## Graphical abstract

An optimized approach toward high output performance for the dielectric elastomer generator without the need for external power supply. Based on the alternating model, the maximum charge density and the maximum energy density can reach 26 mC m^−2^ and 140 mJ g^−1^ per mechanical cycle. Due to all‐solid‐state structure, the generator is capable of working stably under high ambient humidity.

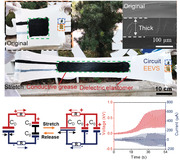

## 1 Introduction

A vast amount of low‐frequency mechanical energy is distributed everywhere, such as waves, wind, and human body movement. Converting these low‐frequency energies into electricity holds great potential for distributed energy, but there still remains challenges, because of the system complexity and frequency inadaptability of traditional electromagnetic technologies.^[^ ^1^ ^]^ In response to this challenge, the past decade has seen a huge effort to develop energy harvesters, especially for lowfrequency energy harvesting.^[^ ^2^, ^3^, ^4^ ^]^

A series of energy harvesters have been developed to convert low‐frequency mechanical energy to electrical energy, such as double‐electrical‐layer energy harvester,^[^ ^5^ ^]^ two‐capacitor electrostatic generator,^[^ ^6^ ^]^ and triboelectric nanogenerator (TENG).^[^ ^7^ ^]^ Core working mechanism of these generators can be classified based on the changing behavior of capacitances.^[^ ^5^, ^6^, ^7^ ^]^ As a notable example, alternating current TENG (AC‐TENG) technology based on contact electrification and electrostatic induction is an emerging new mechanical energy harvesting technology with numerous advantages.^[^ ^7^ ^]^ As a common standard, surface charge density is used to quantify the output performance of TENGs with different structures and different output types.^[^ ^8^ ^]^ Many approaches, such as high‐vacuum^[^ ^9^ ^]^ and ion injection,^[^ ^10^ ^]^ have been carried out to enhance the charge density. However, under the limitation of air breakdown, the maximum charge density only reaches 1.2 mC m^−2^.^[^ ^9^ ^]^ Hence, a new approach to achieving a high charge density was developed to focus on the design of external circuit optimization, such as the charge pumping,^[^ ^11^, ^12^ ^]^ bi‐directional charge shuttle,^[^ ^13^ ^]^ and charge excitation.^[^ ^14^, ^15^ ^]^ However, there is a maximum charge density (2.38 mC m^−2^) that can be achieved at AC‐TENG due to air breakdown between two friction surfaces.^[^ ^15^ ^]^ By contrast, based on contact electrification and electrostatic breakdown effects, direct‐current TENG (DC‐TENG) exhibits a higher charge density (≈8.8 mC m^−2^).^[^ ^16^ ^]^ However, the DC‐TENG works well for rotary mode, especially at a high rotation rate, but they lack the elasticity needed for harvesting large strains.

Dielectric elastomer generator (DEG) with high dielectric strength is an interesting option to fulfill potential high charge density and offers a soft, lightweight, ecologically friendly, and economical solution. DEG is composed of two basic elements: a dielectric elastomer capacitor (DEC) and an external bias voltage.^[^ ^17^ ^]^ The DEC (*C*) is a dielectric elastomer thin film sandwiched between two deformed electrodes. The external bias voltage (*V*) is used to inject charge (*Q*) into the DEC. Mechanically varying the DEC area, and thus the capacitance of the DEC, produces a voltage change according to *Q = CV*, which enables charge storing in the DEG to unidirectionally flow to external load in the form of direct current (DC). The transferred charge is consumed or stored by an external load. On the next cycle, the charge consumed will be re‐supplemented by the external bias voltage. Meanwhile, DEG exhibits high energy density up to hundreds of mJ g^−1^ per cycle,^[^ ^18^, ^19^, ^20^, ^21^ ^]^ but the required external bias voltage is in the kilovolt range, and a practical application is still limited. To eliminate this dependence on the high external bias voltage, various DEGs, e.g., contact‐separation DEG,^[^ ^22^, ^23^ ^]^ hybrid piezoelectric‐DEG,^[^ ^24^, ^25^ ^]^ and self‐prime DEG^[^ ^24^, ^26^, ^27^ ^]^ have been developed. However, due to structural limitations and material limitations, their motion is restricted to a single degree of freedom.^[^ ^28^ ^]^ More importantly, energy density of these generators remains substandard level (<10 mJ g^−1^ per cycle), which makes them hard to compete with the traditional DEG or other energy harvesting technologies.

Here, we design a dielectric elastomer nanogenerator (DENG) to overcome the disadvantages of the DEG with the retention of all the advantages. An electret electrostatic voltage source (EEVS) and a charge‐pump circuit are used to get rid of external high‐power supply and to obtain high voltage. The stretchability of the DEC, as the only active part, enables the DENG to take a variety of shapes and enables multi‐degree‐of‐freedom in movement. Based on shuttle movement of internal charges in the DENG, the AC method is employed to harvest energy. Further, based on the AC method, the energy density and the charge density of the DENG with the thickness of 100 µm reach 140 mJ g^−1^ and 26 mC m^−2^, respectively, by modulating the individual capacitor and increasing the capacitance change of the DEC. When normalized to the volume of dielectric elastomer, the charge density of the DENG reaches 2.5 × 10^8^ mC m^−3^. The charge density of the DENG, to the best of our knowledge, is greater than all the previously reported TENGs. Moreover, a wave energy harvester (WEH) was fabricated based on the DENG, and a self‐powered remote temperature reading system was demonstrated.

## 2 Results and Discussion

### 2.1 Structural Design and Working Mechanism of DENG

**Figure 1:**
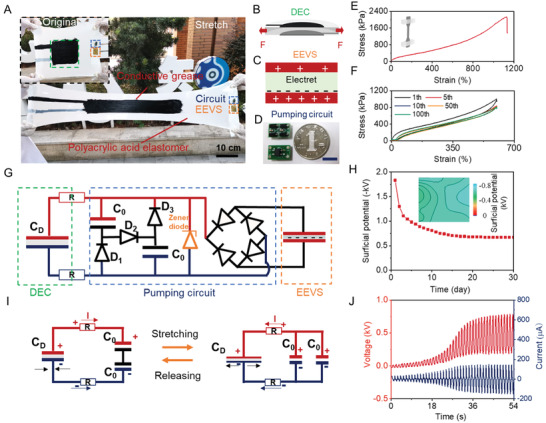
Device structure and working mechanism. A) Photographs of DENG at original state (inset) and stretched state. B) Structural schematic of DEC. DEC is a soft capacitor, made of a dielectric elastomer film sandwiched between two compliant electrodes. C) Structural schematic of electret electrostatic voltage source. D) Photograph of the front and back sides of the pump circuit, next to a Chinese 1‐yuan coin. E) Uniaxial tensile test of the DEG. F) Stress–strain curve of the elastomer in cyclic stress–strain test (600% strain) under successive stretching. Sample width, 10 mm; thickness, 0.1 mm; gage length, 40 mm. Stretching speed, 50 mm min^−1^. G) Circuit diagrams of the DENG. This DENG consists of three parts: DEC, pump circuit, and EEVS. H) Surface potential decay of the electret PTFE film with negative charges. The inset: surface potential image. I) Schematic illustration of AC method working mechanism. J) Output voltage and current curve of the DENG (operation frequency: 0.8 Hz).

As shown in **Figure** **1**A, a DENG is composed of three parts: a DEC (Figure 1B), an EEVS (Figure 1C), and a charge‐pump circuit (Figure 1D). As the only active part, the DEC shares the structure of the conventional DEG with dielectric elastomer (≈100 µm thick) sandwiched between two compliant grease electrodes (Figure S1, Supporting Information). The dielectric elastomer endows the DEC with superior flexibility. Uniaxial tensile tests are performed to evaluate the mechanical properties of the DEC (inset in Figure 1E). As shown in Figure 1E, the uniaxial stretch shows 1100% strain and high tensile (≈2 MPa). Meanwhile, the DEC is soft (elastic modulus ≈20 kPa) and exhibits low hysteresis elasticity for 100 cycles of 600% strain after the first loading cycle (Figure 1F).

The EEVS, as an initial charge source, consists of a charged PTFE electret film and two electrodes (Figure 1C). After the corona polarization treatment, an initial surface potential distribution of ≈−1.01 kV can be obtained and then kept stable at ≈−0.55 kV on the surface of the PTFE film (Figure 1H and Figure S2, Supporting Information). Theoretically, the charge can be retained for hundreds of years,^[^ ^29^ ^]^ which guarantees the sustainable active status of the EEVS for real applications.^[^ ^30^ ^]^ Based on electrostatic induction, a voltage drop is induced by the difference in the charge distribution between two electrodes. When the DEC is connected to the EEVS through a rectifier, an initial voltage of ≈8.6 V can be obtained (Figure S3, Supporting Information). When the DEC is stretched, the capacitance of the DEC increases, resulting in the reduction of the voltage to ≈6 V (Figure S3C,D, Supporting Information). However, the voltage is too low for energy harvesting. Thus, to obtain the high voltage of the DEC, the DENG adopts the charge‐pump circuit connected in parallel to the DEC, as shown in Figure 1G. The pump circuit consists of a self‐prime circuit and a Zener diode (Figure 1D). The self‐prime circuit, which consists of two commercialized capacitors and three diodes arranged in branches, is used to boost voltage output. The Zener diode is used to stabilize the voltage to avoid the dielectric elastomer film breakdown by releasing the surplus charges.^[^ ^14^, ^27^ ^]^ Inspired by alternative‐current nanogenerators,^[^ ^5^, ^6^, ^7^, ^13^ ^]^ external loads, or rectifiers, are placed between the DEC and the pump circuit for harvesting energy (Figure 1G,I and Figure S4A, Supporting Information). The three components of the DENG are displayed as building blocks to maintain the high flexibility, as shown in Figure 1G.

To validate the working mechanism of the DENG, we used two equivalent circuits to describe AC process shown in Figure 1I. The DEC and the circuit are represented using the capacitor C_D_ and two individual capacitors C_0_, respectively. Two resistors represent external loads. As is well known, the capacitance (*C*_D_) of the DEC changes, thus voltage (*V*_D_) of the DEC, inducing a voltage difference between the DEC and the charge‐pump circuit. Therefore, during the stretching process, if the *V*_D_ is lower than the voltage of any individual capacitor, the diode *D_1_* and *D_3_* will conduct, and therefore two individual capacitors will connect in parallel. The partial charges stored on the circuit will be transferred from the circuit to the DEC, as shown on the right of Figure 1I. Conversely, during the releasing process, if *V_D_* is higher than the sum of the voltage on two individual capacitors in the circuit, the diode will cause *D_2_* conduct and therefore two capacitors will be connected in series. The partial charges stored on the DEC will be transferred from the DEC to the circuit, as shown on the left of Figure 1I. That is, two individual capacitors in the circuit are in parallel and delivering charges to the stretched DEC, or in series for receipt of charges from the released DEC. Moreover, because the capacitance of the circuit in parallel and in series are 2*C_0_* and *C_0_/2*, respectively, the circuit can also be considered as a variable capacitor. As a cyclic stretch, the current periodically reverses direction and changes its magnitude continuously between two variable capacitors (the DEC and the circuit), as shown in Figure 1J. Consequently, the output current of DENG is AC. Through the rectifier and external capacitor, AC can be converted to DC and the generated electricity can be directly stored in the external capacitor. In contrast to the charge transfer of DC method, which is from the DEG to the external loads and the charge is eventually consumed by the external load, the charge of the AC method shuttles between two variable capacitors of the DENG. Thus, based on the AC method, the DENG can continuously harvest ambient environmental energy to electric energy and does not require re‐voltage‐boosting or re‐supply charge from the external supply. Meanwhile, the diode D_2_ arrangement allows the current through it to separate positive and negative charge carriers (charge generation) and withstands charge separation state to block positive and negative carries flowing back (charge combination). Therefore, during the stretching‐relaxing, the total amount of charges in the DENG rises with the number of switches increases. In order to avoid the breakdown of the dielectric elastomer, a Zener diode is used to release the surplus charge and stabilize the voltage. Thus, as shown in Figure 1j, the working process of DENG can be divided into two stages: voltage‐boosting stage and voltage‐stabilization stage. More details of the voltage‐boosting working mechanism can be found in Figures S5 and S6 (Supporting Information) and Note S1 (Supporting Information).

### 2.2 Optimization of Structural Parameters

To test the output performance of the DENG, four actuating motors are used to drive both equiaxial stretch motions of the DEC of the DENG for accurate and repeatable motion control (Figure S4B,C, Supporting Information). Two capacitors in the pumping circuit are of equal capacitance. The strain is applied with sinusoidal pattern with the frequency of 0.8 Hz. During an electromechanical cycle, the output performance of the DENG is assessed using two main parameters: output energy density (Δ*E*) and transferred charge density (*Q*) with voltage stability. For the output energy of the AC method calculated, the discharging process between two variable capacitors needs to be considered. The output energy Δ*E* of the DENG can be calculated in an electromechanical cycle through Equation (1):

(1) $$\Delta E\;=\frac{1}{2}\;\left(C_{Dmin}+\frac{1}{2}C_{0}\right)V_{1}^{2}-\frac{1}{2}\left(C_{Dmax}+2C_{0}\right)V_{3}^{2}$$

where *C_0_* is the capacitance of the individual capacitor in the pump circuit, *C_Dmin_* and *C_Dmax_* represent the capacitance of the DEC before stretching and after stretching, respectively. *V_1_* and *V_3_* represent the maximum and minimum voltages of the DENG, respectively (Figure S7 and Note S2, Supporting Information). The charge density equals the amount of charge output divided by the initial area per mechanical cycle. Unless otherwise noted, DEC with an initial area of 10 cm^2^ is adopted for the performance test. Moreover, the energy density equals energy output divided by the mass of the dielectric elastomer with an initial area of 10 cm^2^.

**Figure 2:**
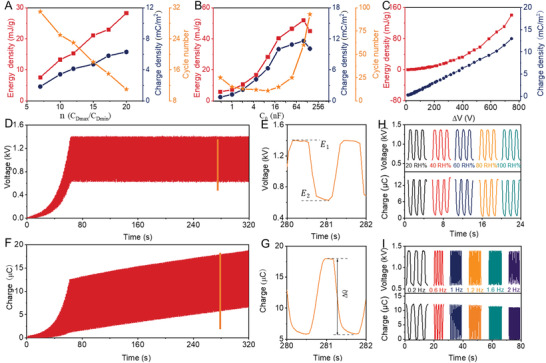
Electric output of DENG with optimized structural parameters. Output performance and voltage‐boosting cycle number with A) different capacitance change ratio *n* of the DEC and B) different individual capacitor *C_0_* under the saturating voltage of 1.16 kV. C) Output performance with different voltage drops. D) Dynamic output voltage and F) dynamic output charge under the saturating voltage of 1.4 kV. E) Enlarged output voltage and G) enlarged output charge. Output voltage and output charge at H) different ambient humidity and at I) different frequency under the saturating voltage of 1.4 kV. Initial area: 10 cm^2^.

The output performance of the DENG strongly relies on capacitance variation of the DEC device during an electromechanical cycle (Figure S8, Supporting Information). The capacitance change ratio can be computed using *n* = *C_Dmax_*/*C_Dmin_*. The change ratio *n* is almost saturated at over 20 under biaxial stretching. In this experiment, the initial capacitance of the DEC, which is also the minimum capacitance (*C_Dmin_*), is 0.58 nF. The individual capacitance (*C*_0_) in the pump circuit is 10 nF. The energy density and the charge density with different change ratios under voltage‐stability state of 1.16 kV and *C*_0_ = 10 nF are given in **Figure** **2**A and Figure S9C (Supporting Information). As *n* becomes larger, the energy density and the charge density increase. Meanwhile, higher *n* implies fewer cycle numbers to reach voltage stability (≈1.16 kV). The trend of experimental results is consistent with that of theoretical calculations (Figure S9A, Supporting Information ). When *C_D_* changes from 0.58 to 11.6 nF, only ten consecutive voltage‐boost cycles reach the saturated voltage of 1.16 kV. The energy density and the charge density reach 28 mJ g^−1^ and 6.3 mC m^−2^ per cycle at 0.8 Hz, respectively. However, due to the limitations in the tensile strength of the dielectric elastomer, it is difficult to further increase the capacitance change of the DEC resulting in higher output performance.

To further optimize the output performance, the influence of the individual capacitor (*C_0_*) in the pump circuit on the output performance and voltage‐boosting cycle number is also investigated (Figure 2B and Figure S9D, Supporting Information). The voltage and the charge density exhibit ascending then descending trend with the increase of *C_0_*. Remarkably, at *C_0_* = 100 nF, only tens of consecutive voltage‐boost cycles reach the saturated voltage (≈1.16 kV). Meanwhile, the output energy density and the charge density approach 52 mJ g^−1^ and 11.2 mC m^−2^ per cycle, respectively (Movie S1, Supporting Information). Nevertheless, when *C_0_* exceeds 100 nF, the output performance of the DENG slightly decreases, which is not consistent with that of theoretical calculations (Figure S9B, Supporting Information). This may be attributed to charge leakage of the individual capacitors. Individual capacitors are the most common commercial polyester capacitors, which are made out of two pieces of plastic film covered with metallic electrodes, wound into a cylindrical‐shaped winding. To increase the capacitance of the capacitor, the area of the plastic film will be increased. With the increase of the area, the number of paths for electrons to flow through the dielectric, and the final result is the increase in the leakage current. When decreasing the thickness of the plastic film increase the capacitance, the result is the same for increasing the area. That is, under the same conditions, charge leakage tends to increase with the increase of the capacitance. Meanwhile, the higher *C_0_* implies the higher the number of cycles and the longer the time for reaching the saturating voltage (≈1.16 kV), which is consistent with that of the theoretical calculation.

Higher voltage leads to higher voltage drop, and thus higher output. The output performance of the DENG visibly elevates with the voltage drop (Figure 2C and Figure S10A, Supporting Information). Finally, the maximum charge density of 13 mC m^−2^ and the maximum energy density of 140 mJ g^−1^ occur before the dielectric elastomer breakdown (Figure 2C and Figure S10B, Supporting Information). On account of the influence of the dielectric breakdown, it is difficult to further improve the output performance of the DENG by enhancing saturating voltage. According to these results above, the output performance could be enhanced by proper selection of the statured voltage, capacitance change ratio *n* of the DEC, and the individual capacitor *C_0_*. In turn, to avoid the dielectric breakdown and obtain high output performance, an exactly matched Zener diode is used to realize the stable operation of the DENG under a high saturating voltage of 1.4 kV (Figure 2D, Detailed descriptions can be found in Materials and Methods). At *C_0_* = 100 nF, with the change of *C_D_* from 0.58 to 11.6 nF, 50 consecutive voltage‐boost cycles reach the saturating voltage of 1.4 kV. Meanwhile, the output energy density and the charge density reach 81 mJ g^−1^ and 12 mC m^−2^ per cycle at 0.8 Hz, respectively, as shown in Figure 2D–G. The power out of the DENG under different resistances is also tested. Under the low stretching frequency of 0.8 Hz, a high maximum peak power of 40 mW is obtained at a resistance of 50 MΩ (Figure S11A, Supporting Information). Meanwhile, the charging curves of different capacitors are also shown in Figure S11B (Supporting Information). It is clear that with smaller capacitance, a higher charge speed would be got. For capacitance of 47 µF, the charging voltage reaches 6 V within 45s.

In addition, the effects of the humidity and frequency on the performance of the DENG are also investigated, as shown in Figure 2H,I and Figure S10 (Supporting Information). Significantly, different from the TENG,^[^ ^31^ ^]^ the output performance of the DENG remains stable from 20 RH% to 100RH % owing to all‐solid‐state structure without air gap (Figure 2H and Figure S10C, Supporting Information). In addition, on account of the influence of the hysteresis elasticity of the dielectric elastomer film, the output performance of the DENG varied slightly with increasing stretching frequency (Figure 2I and Figure S10D, Supporting Information).

### 2.3 Comparison of Output Performance

**Figure 3:**
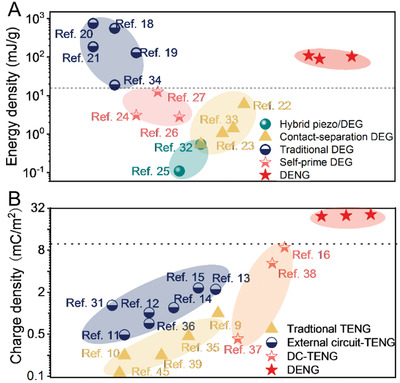
Comparisons of A) energy density with different types of DEGs^[^ ^18^, ^19^, ^20^, ^21^, ^22^, ^23^, ^24^, ^25^, ^26^, ^27^, ^32^, ^33^, ^34^ ^]^ and B) charge density with different types of TENGs per cycle.^[^ ^9^, ^10^, ^11^, ^12^, ^13^, ^14^, ^15^, ^16^, ^31^, ^35^, ^36^, ^37^, ^38^, ^39^, ^45^ ^]^

In **Figure** **3**, we summarize the reported energy density in various DEGs and the charge density in various TENGs, and compare them with our results. With the unremitting efforts of researchers in recent years, various DEGs have been developed (Figure 3A). Based on periodic contact and separation between electret film and the elastomer film, the energy density of contact‐separation DEGs can reach 6 mJ g^−1^ per cycle.^[^ ^22^, ^23^ ^]^ Based on piezoelectric nanogenerator as the charge source, the energy density of piezo‐DEGs can reach 0.285 mJ g^−1^ per cycle.^[^ ^25^ ^]^ Based on the self‐priming circuit and a low voltage source, the self‐priming DEG can passively boost the DEC voltage form low to the kilovolt range over a number of cycles and the energy density can reach 12.6 mJ g^−1^ per cycle.^[^ ^24^, ^26^, ^27^ ^]^ As a comparison, the energy density of DENG substantially surpasses the output of all the DEGs with low bias voltage and without bias voltage, and reaches the same level of traditional DEG with a high external bias voltage of several kilovoltages. Meanwhile, similar to the charge‐shuttling structure,^[^ ^13^ ^]^ positive and negative charges simultaneously shuttle between the DEC and the pump circuit (Figure S12A,B, Supporting Information). The actual charge output ought to be doubled by two mirror charge carries instead of one (Figure S12C,D and Movie S2, Supporting Information). The detailed description is provided in Note S3 (Supporting Information). Therefore, in this work, the maximum actual charge density of 26 mC m^−2^ is over two orders of magnitude larger than that of the stretching‐nanogenerator reported,^[^ ^40^ ^]^ and more than three times than that of a variety of TENGs ever reported^[^ ^16^ ^]^ (Figure 3b, Supporting Information). In addition, when normalized to dielectric elastomer volume (volumetric charge density), the DENG shows the volumetric charge density of 2.5 × 10^8^ mC m^−3^, which is nine orders of magnitude larger than those ever reported 9 mC m^−3^.^[^ ^41^ ^]^

### 2.4 Application Demonstrations of DENG

**Figure 4:**
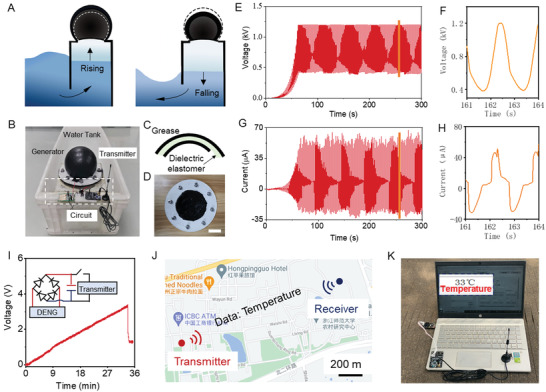
Illustration of the self‐powered remote temperature reading system. A) Schematic illustration of the WEH. The water column falls and rises periodically as water waves, causing the DEC to inflate and deflate in cycles. B) Photo image of the intergraded device of WEH and remote temperature transmitting circuit. C) Structural schematic and D) optical photograph of the DEC amounted on the OWH. Scale bar: 6.5 cm. E) Output voltage and G) output current of WEH at an agitation frequency of 0.5 Hz. F) Enlarged output voltage and H) enlarged output current of WEH at an agitation frequency of 0.5 Hz. I) Voltage variation of the capacitor (2.2 mF) in consequent remote temperature reading system processes. J) The schematic of the distance temperature transmission of the communication nodes on the ZJNU campus using the wave energy. K) Receiver node of self‐powered remote temperature reading system. A computer interface showing the successful transmission of the temperature information.

With high output performance, the DENG is expected to be a fundamental component for developing practical self‐powered systems and energy harvesters. We demonstrate this approach by constructing a WEH (Figure S13A, Supporting Information). The WEH includes an oscillating water column device (OWC) and a DENG. The OWC is composed of a chamber with a water column that is connected to the open wavefield through a large opening in the bottom part and topped by an air chamber in the upper part.^[^ ^42^, ^43^ ^]^ The active part is the DEC mounted onto the top of the air chamber by a clamp with a central circular aperture (**Figure** **4**B–D). The incident waves excite the water in the column and produce its oscillating motion in the vertical direction leading to an alternating compression and decompression of the air in the chamber (Figure 4A). Such pressure variation, in turn, drives inflation and deflation of the DENG and produces electricity (Figure S13B, Supporting Information).

In order to quantify the output performance of the WEH, the device was tested on a wave tank equipment, which was built to simulate real ocean waves in the laboratory stage. To balance the voltage‐boosting number and simplification of the pump circuit, we adopt the capacitor of 18 nF as the individual capacitor of the pump circuit. At the same time, to avoid the dielectric elastomer breakdown, a Zener diode is used to stabilize the saturating voltage at 1.2 kV. The DEC is pre‐inflated to decrease the thickness of the dielectric elastomer and increase the initial capacitance of the DEC. The capacitance change of the DEC was measured from initial capacitance (5 nF) to expanded capacitance (20 nF), indicating a capacitance change rate of ≈4 (Figure S13B, Supporting Information). Based on the wave tank experiment, the output performance of the WEH in water waves was characterized, as shown in Figure 4E–H. After ≈31 cycles, the output voltage is boosted to a high level approaching 1.2 kV and keeps relatively stable. With an agitation frequency of 0.5 Hz, the peak current of ≈50 µA was obtained, and the corresponding charges by integrating the current reach 12.6 µC per cycle (Figure 4H). The maximum peak power of 9.41 mW was obtained with the load resistance of 7 MΩ (Figure S13C, Supporting Information).

The changing climate, with associated higher probability of unexpected severe weather, can damage or destroy vulnerable infrastructure, and result in both economic and human losses.^[^ ^44^ ^]^ Thus, establishing a self‐powered and distributed system for forecasting extreme weather is necessary. To approach such a challenge, the WEH was selected for constructing practical self‐powered remote temperature reading system using water waves as a power source (Figure 4B). The circuit diagram is demonstrated in Figure S14 (Supporting Information). A storage capacitor of 2.2 mF was charged to 3.3 V within 32 min (Figure 4I), then the communication module could be driven. In this process, the released electricity from the capacitor would supply the temperature detection and transmitter. The stable transmitting distance between the transmitter and receiver is demonstrated as far as 1.7 km, as shown in Figure 4I–K and Movie S3 (Supporting Information).

## 3 Conclusions

In summary, a DENG, which is constructed by a DEC, an EEVS, and a charge‐pump circuit, is designed to achieve high performance without the need for any power supplies or generators. The DEC, as the only active part, ensures the multi‐degree‐of‐freedom of the DENG. The EEVS provides the stable initial charge as the ignition charge source. The pump‐charge circuit separates positive and negative charge carriers to improve and stabilize the voltage. The AC method is constructed between the DEC and the circuit and used to improve the output performance. Eventually, by adjusting the individual capacitor and the capacitance change of the DEC, the maximum energy density of 140 mJ g^−1^ and the maximum charge density of 26 mC m^−2^ can be obtained based on alternating charge movement. Meanwhile, the DENG showed high stability with the humility changing from 20 RH% to100 RH% and frequency stability changing from 0 to 2 Hz. Furthermore, the DENG can be integrated into a WEH, thereby converting wave energy to electrical energy. The electrical energy generated by the WEH is sufficient to charge a commercial capacitor and to power a remote temperature reading module.

## 4 Experimental Section

### Fabrication of the EEVS

The PTFE film (thickness of 50 µm) was charged in air condition with the assistance of metal grid, and the metal grid was 8 cm away from the PTFE film (Figure S2, Supporting Information). A bottom electrode and a top PTFE film coated with Au electrode constructed an EEVS. The surface potential was tested using an electrostatic voltmeter (Model 347, TRek).

### Preparation of the DEC

The two‐side of the dielectric elastomer (70 410, Tesa) were coated by conductive grease (KS‐660, Shin‐Etsu). The thickness and density of the elastomer are 100 µm and 1 g cm^−3^, respectively. The EEVS and other parts were electrically connected through a rectifier (KBJ1510). The self‐prime circuit was fabricated using three rectifier diodes (1N4007) and two commercial polyester capacitors. The diode (1N4007) and the Zener diodes (1N5387B) were used between positive and negative pins of the pump circuit to avoid dielectric breakdown. Different saturating voltages could be achieved via modulation of the combination of the rectifier diode (1N4007) and the Zener diodes (1N5387B) in series.

### Characterization of the Device

The mechanical characteristics of the dielectric elastomer were determined using a tensile test by an electronic universal material testing machine (Zwicki, Zwick, German). The surface potential of PTFE film was mapped by a high‐speed electrometer (Model‐347, Trek, America). The output voltage of the DENG was measured by a high‐speeded electrometer (Model‐344, Trek, America). The periodic stretching‐releasing process of the DEC was stimulated by four actuating motors (86BYG250E, Yixing, China), which were controlled by a motion control system (TC5520, Topcnc, China). The charges, the short current, and the voltage of the capacitor were measured with a programmable electrometer (6514, Keithley). The cross‐section morphology of the dielectric elastomer was probed by a high‐resolution field emission scanning electron microscope (Nova NanoSEM 450, FEI). The capacitance of the DEC was measured with the impedance analyzer (IM3523, HIOKI). Wave maker (CP‐65, Jebao) was used to generate water waves in wave tank with a dimension of 1.5 × 0.37 × 0.27 m. The OWH is 27 cm high by 22 cm width. The opening in the bottom part is 10 cm tall by 22 cm width.

## Conflict of Interest

The authors declare no conflict of interest.

## Supporting information

Supporting Information

Supplemental Movie 1

Supplemental Movie 2

Supplemental Movie 3

## Data Availability

Research data are not shared.
