# Supplementary material for: High‐Performance Dielectric Elastomer Nanogenerator for Efficient Energy Harvesting and Sensing via Alternative Current Method
Source: Adv Sci (Weinh). 2022 Apr 9;9(18):2201098. doi: 10.1002/advs.202201098 (PMC9218771; doi:10.1002/advs.202201098)
Supplement: Supplementary file 1 — Supporting Information [file ADVS-9-2201098-s003.pdf]

ADVANCED SCIENCE
Open Access

Supporting Information

for *Adv. Sci.*, DOI 10.1002/advs.202201098

High-Performance Dielectric Elastomer Nanogenerator for Efficient Energy Harvesting and Sensing via Alternative Current Method

Zisheng Xu, Kunwei Bao, Kui Di, Haojie Chen, Jianbo Tan, Xinjun Xie, Yixin Shao, Jiancheng Cai, Shizhe Lin, Tinghai Cheng, Shiju E, Kang Liu* and Zhong Lin Wang**

© 2022 Wiley-VCH GmbH.

Supporting Information

Title

High-performance Dielectric Elastomer Nanogenerator for Efficient Energy Harvesting and Sensing via Alternative Current Method

Zisheng Xu, Kunwei Bao, Kui Di, Haojie Chen, Jianbo Tan, Xinjun Xie, Yixin Shao,

Jiancheng Cai, Shizhe Lin, Tinghai Cheng, Shiju E, Kang Liu*, Zhong Lin Wang**

Supplementary Note 1: Theoretical analysis of the DENG

Due to the inherent properties of materials, the capacitance of the MLCC capacitors varies in a nonlinear fashion with the external voltage. Thus, to reasonably evaluate the performance of the DENG, commercial non-polar capacitors were used to analyze and optimize the performance of the DENG. At different points, C_{Dx}, V_{Dx}, and Q_{Dx} are used to represent the capacitance, the voltage, and the total charge of the DEC, respectively;C_{Sx}, V_{Dx}, and Q_{Sx} are used to represent the capacitance, the voltage, and the total charge of the pump circuit, respectively; C_x, V_x, and Q_x are used to represent the capacitance, the voltage, and the total charge of the DENG, respectively. For the pump circuit with two same capacitors, the capacitance values in series and in parallel are given by $C_S = \frac{C_0}{2}$ and $C_s' = 2C_0$, respectively. The subscript x is used denote the point index. To simplify the derivation, leakage losses will be ignored. A theoretical model on the behavior of the DENG is established.

To better understand the process of charging shuttling and charge pump process, a test based on biaxial stretching is discussed in detailed. An EEVS is connected to the circuit and charges flows into DEG and the pump circuit in series through D_2, as depicted in Fig. 1. A cycle of the voltage-boost process can be divided into four sub-stages: 1-2, 2-3, 3-4, and 4-5, as is shown in Fig. S5. Initially, the DEC is unstretched. the capacitance and the voltage of the DEC appears as C_{Dmin} and V_{D1} at point 1.

1-2: When the DEC is stretched, the capacitor (C_D) of the DEC rises while the voltage (V_D) of the DEC decreases, inducing a voltage difference between the DEC and the pump circuit in series. The electrical performance of the DENG can be expressed according to the following equations.

At point 1, the voltages of the DEC and the pump circuit are equal:

$$V_{D1} = V_{s1} \tag{1}$$

Under unstretched state, the capacitance of the DEC is at the minimum value $C_{D_{min}}$:

$$C_{D1} = C_{D_{min}} \tag{2}$$

The charge in the DEC is given by:

$$Q_{D1} = C_{D_1}V_{D1} = C_{D_{min}}V_{D1} \tag{3}$$

Due to $V_{D1} = V_{S1}$, the charge Q_{S1} in the pump circuit is：

$$Q_{S1} = C_{S1}V_{S1} = C_{S1}V_{D1} \tag{4}$$

Due to the pump circuit in series ($C_{S1} = C_0/2$), the charge Q_{S1} of the pump circuit:

$$Q_{S1} = \frac{C_0V_{D1}}{2} \tag{5}$$

According to Supplementary Equation 2-4, at point 1, the total charge Q_1 in the DENG can be described:

$$Q_1 = Q_{D1} + Q_{s1} = C_{Dmin}V_{D1} + \frac{C_0 V_{D1}}{2} \tag{6}$$

Notably, during the process from point 1 to point 2, as the diode D_2 is reverse biased, no charges flow through two loads from the pump circuit to the DEC. Therefore, the total charge amount of the charge is conserved during the process. Until the voltage of the DEC is lower than the voltage of any individual capacitor in the pump circuit, which causes the diodes D_1 and D_3 to conduct, the PUMP circuit switches from series configuration to parallel configuration. Therefore, the point 2 is a critical point. Herein, at point 2, the voltage of the DEC is equal to the voltage of the SPC circuit. Since two individual capacitors in the pump circuit are equal, the voltages of the DEC and the pump circuit are equal and can be expressed:

$$V_{D2} = V_{s2} = \frac{V_{D1}}{2} \tag{7}$$

During the process from point 1 to point 2, no charge flows between the pump circuit and the DEC. Therefore, the charges Q_{D2} in the DEC is equal to Q_{D1}:

$$Q_{D2} = Q_{D1} = C_{D_{min}}V_{D1} \tag{8}$$

Due to the transition of the circuit configuration in the pump circuit from series configuration to parallel configuration ($C_s = 2C_0$), the charge Q_{S2} of the pump circuit is:

$$Q_{S2} = 2C_0 V_{D2} = C_0 V_{D1} \tag{9}$$

According to Supplementary Equation 8-9, the total charge Q_2 in the DENG can be described:

$$Q_2 = Q_{D2} + Q_{S2} = C_{Dmin}V_{D1} + C_0 V_{D1} \tag{10}$$

According to Supplementary Equation 9-10, after the pump configuration transition, the increased charges ΔQ_{1-2} can be expressed:

$$\Delta Q_{1-2} = Q_2 - Q_1 = C_0 V_{D1}/2 \tag{11}$$

2-3: The voltage of the DEC continues to decrease as the DEC is stretched. Charges flow through two loads from the pump circuit to the DEC (Supplementary Fig. S5e). Until the stretch ceased at point 3, the DEC and the pump circuit appear as the maximum C_{Dmax} and parallel configuration $2C_{0,}$ respectively, and the voltages of the DEC and the pump circuit are equal.

At point 3, the charge Q_{D3} of the DEC is:

$$Q_{D3} = Q_{D2} + Q_T = C_{Dmin}V_{D1} + Q_T \tag{12}$$

At the same time, the voltage of the DEC can be obtained:

$$V_{D3} = \frac{Q_{D3}}{C_{\mathrm{Dmax}}} = \frac{Q_{D2}+Q_T}{C_{\mathrm{Dmax}}} \tag{13}$$

The charge Q_{S3} and voltage V_{S3} of the pump circuit are:

$$Q_{S3} = Q_{S2} - Q_T \tag{14}$$

$$V_{S3} = \frac{Q_{S3}}{2C_0} = \frac{Q_{s2}-Q_T}{2C_0} \tag{15}$$

Due to $V_{D3} = V_{S3}$, according to Supplementary Equation 8, 9, 13 and 15, the shuttling charge Q_T between the DEC and the pump circuit can be expressed:

$$Q_T = \frac{C_0V_{D1}(C_{Dmax}-2C_{Dmin})}{C_{\mathrm{Dmax}}+2C_0} \tag{16}$$

It is common to replace the capacitance of the DEC with the ratio of capacitance change of the DEC($n = \frac{C_{Dmax}}{C_{Dmin}}$) and the ratio between the unstretched capacitance (C_{Dmin}) and the individual capacitance of the pump circuit ($\frac{C_0}{C_{Dmin}} = r$):

$$Q_T = \frac{C_0V_{D1}(n-2)}{n+2r} \tag{17}$$

3-4: Conversely, when the DEC is released, the capacitance of the DEC decreases while the voltage of the DEC increases, inducing a voltage difference between the DEC and the pump circuit in parallel. As the diodes D_1 and D_3 are reverse biased, no charges flow through two loads from the DEC to the pump circuit. Until the voltage of the DEC is higher than the voltage of two capacitors in series, which causes the diode D_2 to conduct, the pump circuit switches from parallel configuration to series configuration. Herein, the point 4 is a critical point of the configuration transition, as is shown in Fig. S5d.

During the process from point 3 to point 4, no charge flows between the pump circuit and the DEC, and the charges Q_{D4} in the DEC is equal to Q_{D3}.

Due to $Q_{D4} = Q_{D3}$, the voltage V_{D4} of the DEC can be obtained:

$$V_{D4} = \frac{Q_{D3}}{C_{D4}} = \frac{Q_{D2}+Q_T}{C_{D4}} \tag{18}$$

As the charges of the DEC in two points (3 and 4) are equal, the capacitance C_{D4} of the DEC can be expressed:

$$C_{D4} = \frac{V_{D_3}C_{\mathrm{Dmax}}}{V_{D4}} \tag{19}$$

Due to $V_{D4} = V_{D4} = 2V_{D3}$, the capacitance C_{D4} and the voltageV_{s4} of the DEC can be written:

$$C_{D4} = \frac{C_{\mathrm{Dmax}}}{2} \tag{20}$$

$$V_{S4} = V_{D4} = 2V_{D3} = \frac{2Q_{S4}}{C_0} \tag{21}$$

According to Equation 20-21, the charge Q_{S4} in the pump circuit can be written:

$$Q_{S4} = \frac{C_0V_{D4}}{2} = \frac{C_0(Q_{D2}+Q_T)}{C_{\mathrm{Dmax}}} \tag{22}$$

By combing with Equation 21 and 13, the charge Q_4 in the DENG is given by:

$$Q_4 = Q_{S4} + Q_{D4} = \frac{C_0(C_{Dmin}V_{D1}+Q_T)}{C_{\mathrm{Dmax}}} + C_{Dmin}V_{D1} + Q_T \tag{23}$$

4-5: The voltage of the DEC continues to increase as the DEC is released. Under the voltage difference between them, opposite charges of magnitude flow into two capacitors in series through two loads from the DEC, as is shown Fig. 5c. Meanwhile, conservation of charge requires that charges on two plates of the individual capacitors keep equal-magnitude, and thus the positive and negative charge carries in the D_2 diode separate from each other, compensating for charges of two adjacent plates of two capacitors, respectively. Finally, at the point 5, the DEC returns to its initial state, and the total amount of charge stored in the DENG increases during the stretching-releasing process.

At point 5, the charges in the pump circuit and the DEC are：

$$Q_{S5} = Q_{S4} + Q_T' \tag{24}$$

$$Q_{D5} = Q_{D4} - Q_T' \tag{25}$$

Where Q_T' is shuttling charge the during the process from point 4 to point 5.

Due to $V_{D5} = V_{S5}$, according to S24-25, the shuttling charge from the DEG to the pump circuit can be obtained：

$$Q_T' = \frac{C_0Q_{D4}-2C_{Dmin}Q_{S4}}{2C_{Dmin}+C_0} \tag{26}$$

Q_{D4} and Q_{S4} can be substituted by Equation 12 and 22:

$$Q_T' = \frac{C_0(Q_{D2}+Q_T)-C_{Dmin}\frac{C_0(Q_{D2}+Q_T)}{C_{D4}}}{2C_{Dmin}+C_0} = \frac{C_0(Q_{D2}+Q_T)-C_{Dmin}\frac{2C_0(Q_{D2}+Q_T)}{C_{Dmax}}}{2C_{Dmin}+C_0} \tag{27}$$

By combing $n = C_{Dmax}/C_{Dmin}$:

$$Q_T' = \frac{C_0(1-\frac{2}{n})(Q_T+Q_{D2})}{2C_{Dmin}+C_0} \tag{28}$$

According to Equation 16, we can obtain:

$$Q_T' = \frac{C_0\left(1-\frac{2}{n}\right)\left(\frac{C_0V_{D1}\left(C_{Dmax}-2C_{Dmin}\right)}{C_{\mathrm{Dmax}}+2C_0}+C_{Dmin}V_{D1}\right)}{2C_{Dmin}+C_0} = \frac{V_{D1}(n-2)(C_0+C_{Dmin})}{\frac{2C_{\mathrm{Dmax}}}{C_0}+4+n+\frac{2nC_0}{C_{\mathrm{Dmax}}}} \tag{29}$$

According to Supplementary Equation 16:

$$\frac{V_{D5}}{V_{D1}} = \frac{V_{D5}C_{Dmin}}{V_{D1}C_{Dmin}} = \frac{Q_5}{Q_1} \tag{30}$$

No charges increase anywhere in the DENG during the process from 4 to 5; thus, the charge is the same as at point 5 is at point 4:

$$Q_5 = Q_4 = Q_{D4} + Q_{S4} = Q_{D3} + Q_{S4} \tag{31}$$

The substitute Equation 12 and Equation 22 into the Equation 28:

$$Q_5 = V_{D1}C_{Dmin} + Q_T + \frac{C_0(V_{D1}C_{Dmin}+Q_T)}{C_{Dmax}} \tag{32}$$

Inserting Equation 16 into Equation 29:

$$Q_5 = \frac{V_{D1}(C_0+C_{Dmin})(C_0+C_{Dmax})}{C_{Dmax}+2C_0} \tag{33}$$

Thus:

$$\frac{V_{D5}}{V_{D1}} = \frac{Q_5}{Q_1} = \frac{2(C_0+C_{Dmin})(C_0+C_{Dmax})}{(C_{Dmax}+2C_0)(2C_{Dmin}+C_0)} \tag{34}$$

After a stretching-releasing cycle, the increased charges ΔQ in the DENG can be expressed:

$$\Delta Q = Q_5 - Q_1 = \frac{1}{2}Q_T = \frac{V_{D1}C_0(C_{Dmax}-2C_{Dmin})}{2(C_{Dmax}+2C_0)} \tag{35}$$

Meanwhile, when the charge of the DENG reaches a certain value, the further charge pump would create electric breakdown of the dielectric elastomer. As is shown in Supplementary Fig 6, in the process of the releasing process (4-5), as V_D increases, the electric field of the DENG increases. To avoided the dielectric breakdown, a Zener diode is used to release surplus charges and the DENG reaches a stable state.

Supplementary Note 2: Theoretical analysis of energy density of the DENG

As the DEC was cyclically stretched, capacitances and voltages of DENG were varied between two fixed capacitances (high capacitance C and low capacitance C) and two fixed voltages (high voltage V1 and low voltage V2), respectively. To illustrate the principle of the DENG, a simplified energy harvesting cycle is schematically shown on the voltage-charge plane in Fig. S7b.

The electric energy E stored in a system can be calculated according to the following equation:

$$E = QV = \frac{1}{2}CV^2 \tag{36}$$

Where C, V, and Q is the capacitance, the voltage of the DENG, and charge stored in the DENG, respectively.

At un-stretched state (point 1, $V_1 = V_{D1}$)), the electric energy stored in the DENG can be calculated:

$$E_{Deng1} = E_{D1} + E_{P1} = \frac{1}{2}(C_{Dmin} + \frac{1}{2}C_0)V_{D1}^2 \tag{37}$$

At the maximum stretched state (point 3), the DENG is at a condition of low voltage ($V_3 = V_{D3}$) and high capacitance ($C_{Dmax} + 2C_0$). the electric energy stored in the DENG can be calculated :

$$E_{Deng1} = E_{D1} + E_{P1} = \frac{1}{2}(C_{Dmax} + 2C_0)V_{D3}^2 \tag{38}$$

The output electric energy can be rewritten as:

$$\Delta E = E_{Deng1} - E_{Deng3} = \frac{1}{2}(C_{D1} + \frac{1}{2}C_0)V_{D1}^2 - \frac{1}{2}(C_{Dmax} + 2C_0)V_{D3}^2 \tag{39}$$

Supplementary Note 3: Measuring the charge on both sides

As demonstrated in Supplementary Fig. 12a, the charge output on two sides were measured by two steps, respectively, with an electrometer Keithley 6514. In the first step, the charge out on the positive side was measured by the electrometer. In the second step, the charge output of the opposite side was also measured by the electrometer. As shown in Supplementary Fig. 12b, the charge outputs of both sides are equal. As shown in Supplementary Fig. 12b, to further verify this conclusion, two arrays of 52 white LEDs were placed on two rectifiers of both sides, respectively. When stretching the DEC, two arrays of 50 white LEDs illuminated at the same time.

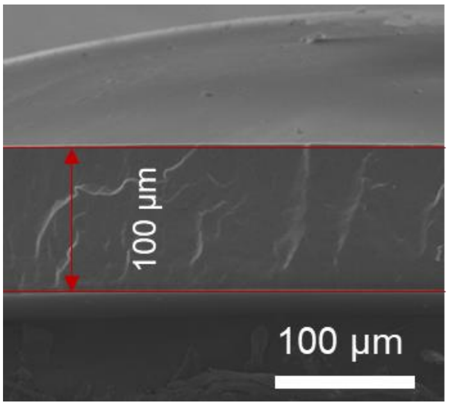

Figure S1. Cross-sectional SEM image of the DE thin film.

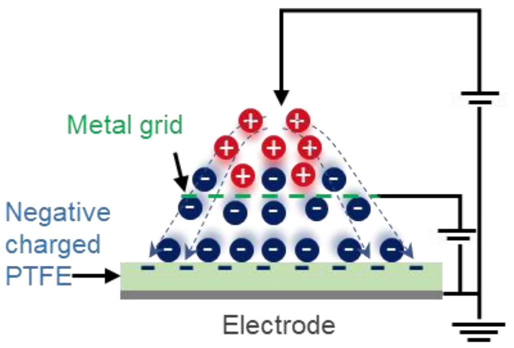

Figure S2. Scheme of the corona discharging

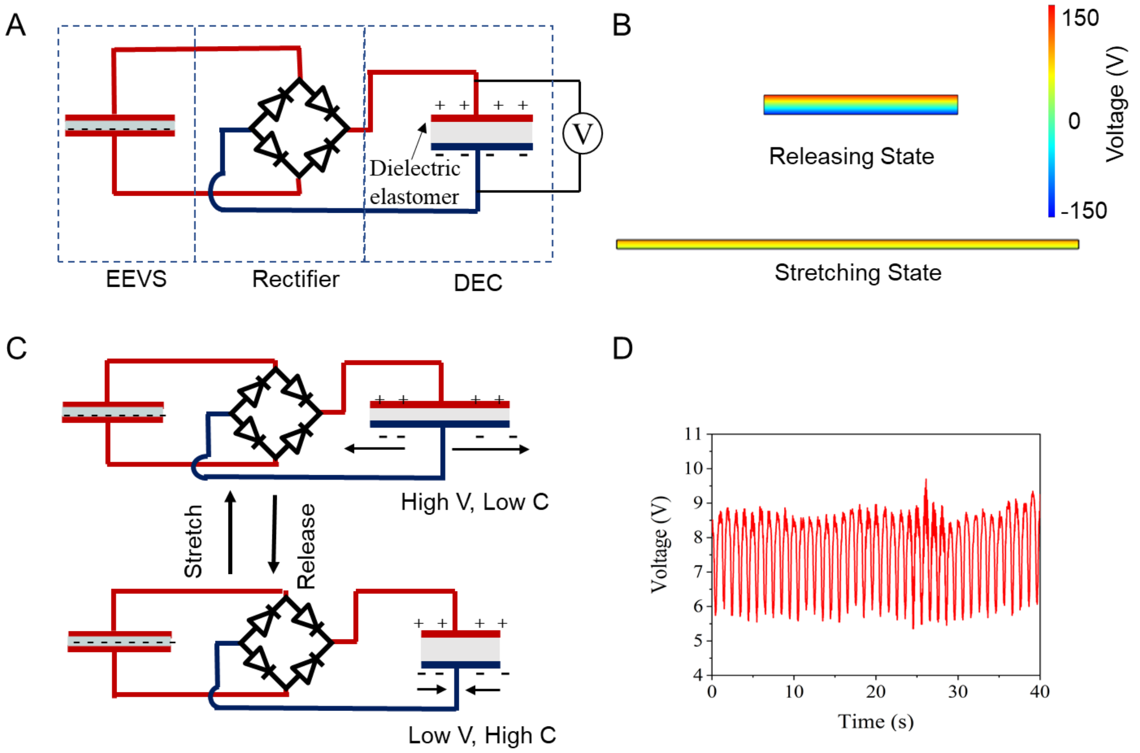

Figure S3 **A** Schematic picture of voltage testing setup by EEVS. **B** Finite-element Simulation result of potential distribution under the open-circuit condition with the change in the thickness of the dielectric elastomer film, and with the charge density of 1 mC/m^2 using COMSOL. Relative dielectric constant: 4; Young modulus: 750 kPa. **C** Mechanism of energy harvesting by stretching the dielectric elastomer. **D** Measured voltage of the DEC under 0.8 Hz stretching.

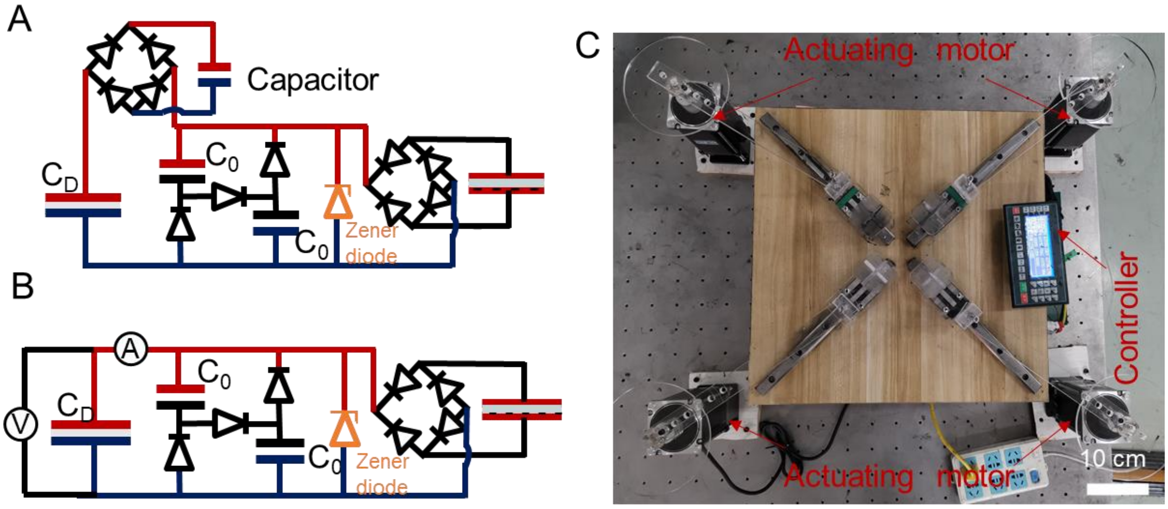

Figure S4 **A** Circuit model of the DENG for energy harvesting. **B** Testing schematic of short-circuit current and output voltage. **C** Programmable biaxial tensile tester.

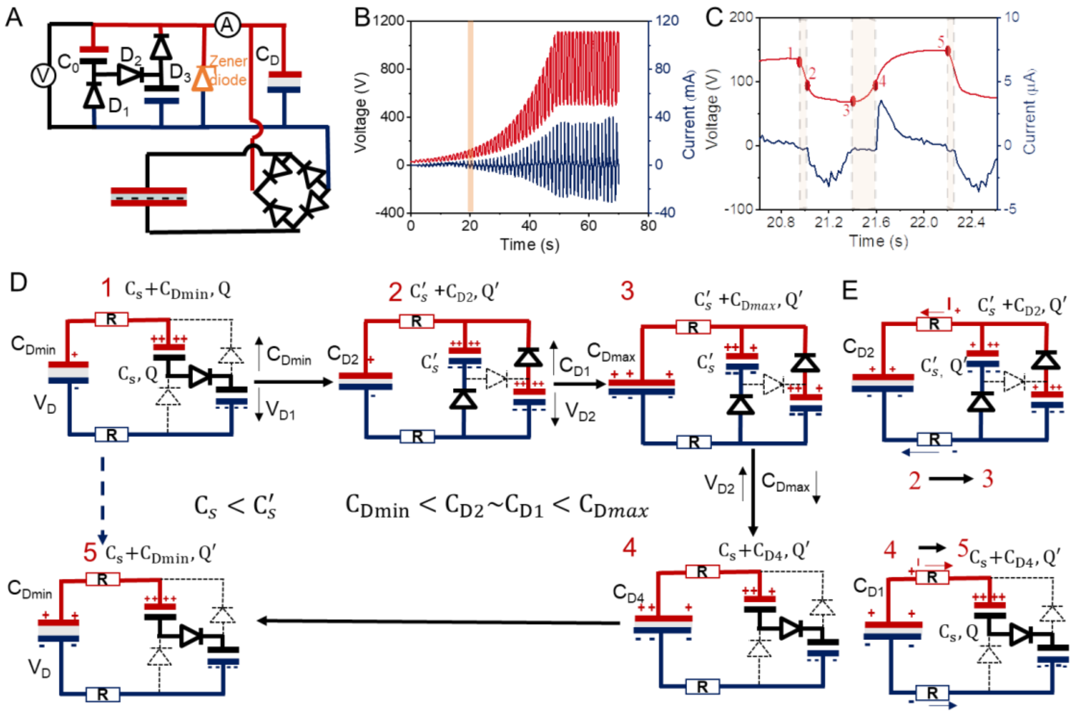

Figure S5 Schematics diagram of the working mechanism of the DENG. A Schematics diagram of measurement circuits for current and output voltage. **B** Voltage and current curves of DENG at 0.8 Hz. The capacitance of the individual capacitor in the pump circuit is 100 nF, and the capacitance of the DEC varies from 0.58 nF to 11.6 nF. **C** Enlarged output voltage and current curves and corresponding working process. **D** Schematic depicting DENG cycle process: Steps 1-3: DEC with stretching; Steps 3-5: DEC with releasing. **E** Schematic depicting charge transfer process cycle process. Steps 2-3: charges transfer from the circuit to DEC; Steps 4-5: charges transfer from DEC to the circuit.

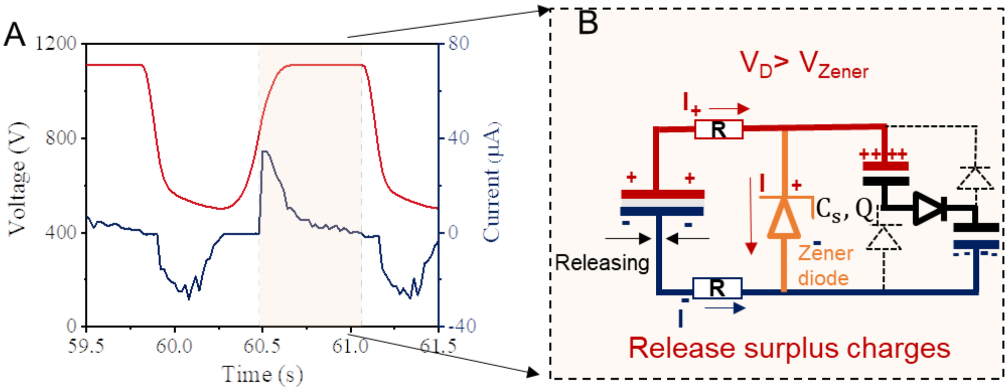

Figure S6 Work principle of DENG with voltage stabilization. A Dynamic current and voltage output of DENG with voltage stabilization (1.2 kV) under 0.8 Hz. **B** Schematic diagram of discharge protection working principle.

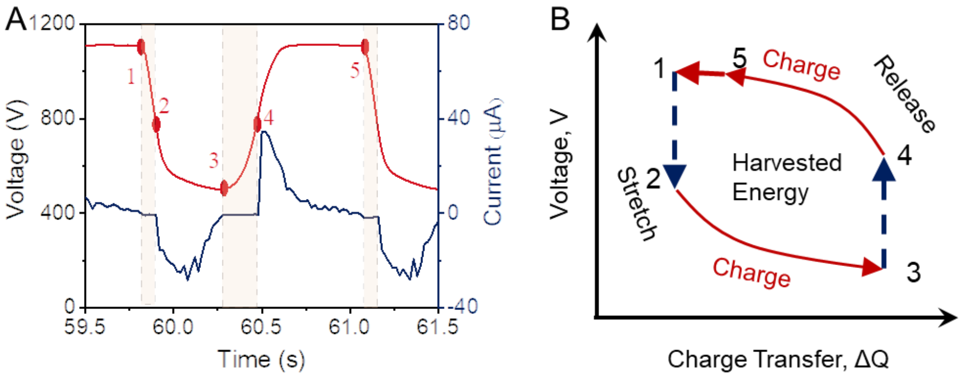

Figure S7 Schematic depicting DENG cycle process with voltage-stabilization. A Enlarged output voltage and current curves at 0.8 Hz with voltage-stabilization and corresponding working process. The specific operation processes are the same as the Fig. S5-6. **B** This process can be represented thermodynamically in terms of voltage and charge in an energy harvesting cycle. The harvested energy is the area enclosed by a cycle with voltage-stabilization.

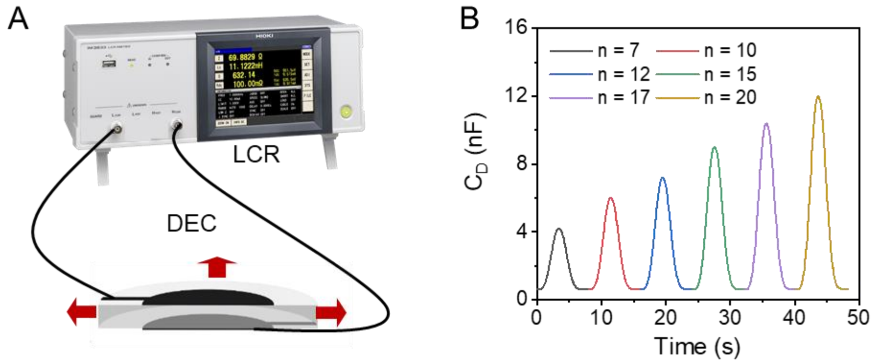

Figure S8 **A** schematic diagram of the capacitance test. **B** Capacitance change responses of the DEC with 10 cm^2 with six different stretching ratios.

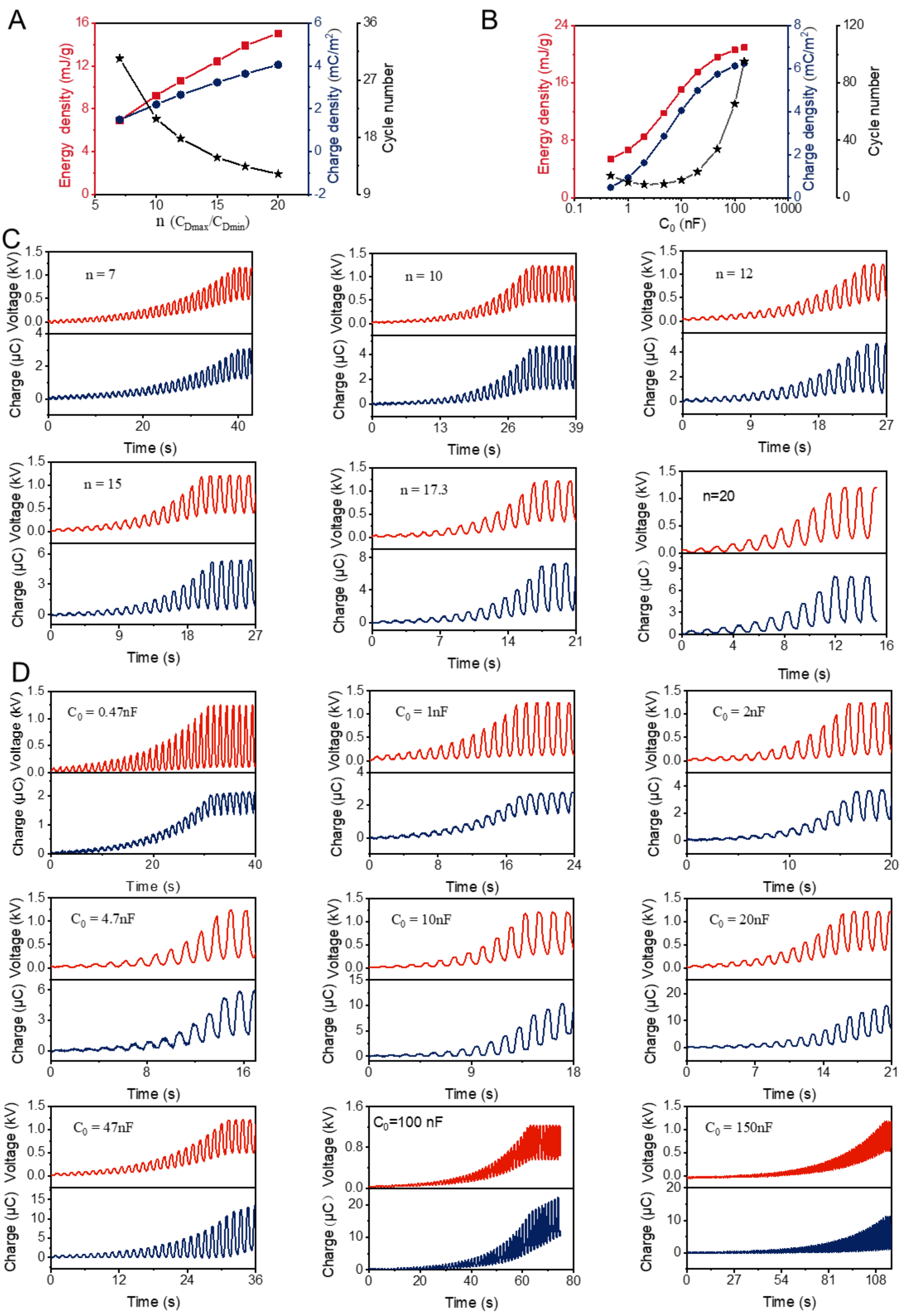

Figure S9 Theoretical energy density, charge output, and voltage-boosting cycle number of the DENG **A** with different capacitance change ratio and **B** with different individual capacitance C_0. Output charge and voltage curve of the DENG with 10 cm^2 **C** at C_0=10 nF with different n and **D** at n=20 with different C_0. Theoretical results can be obtained by Equations 16, 34, 35, and 39.

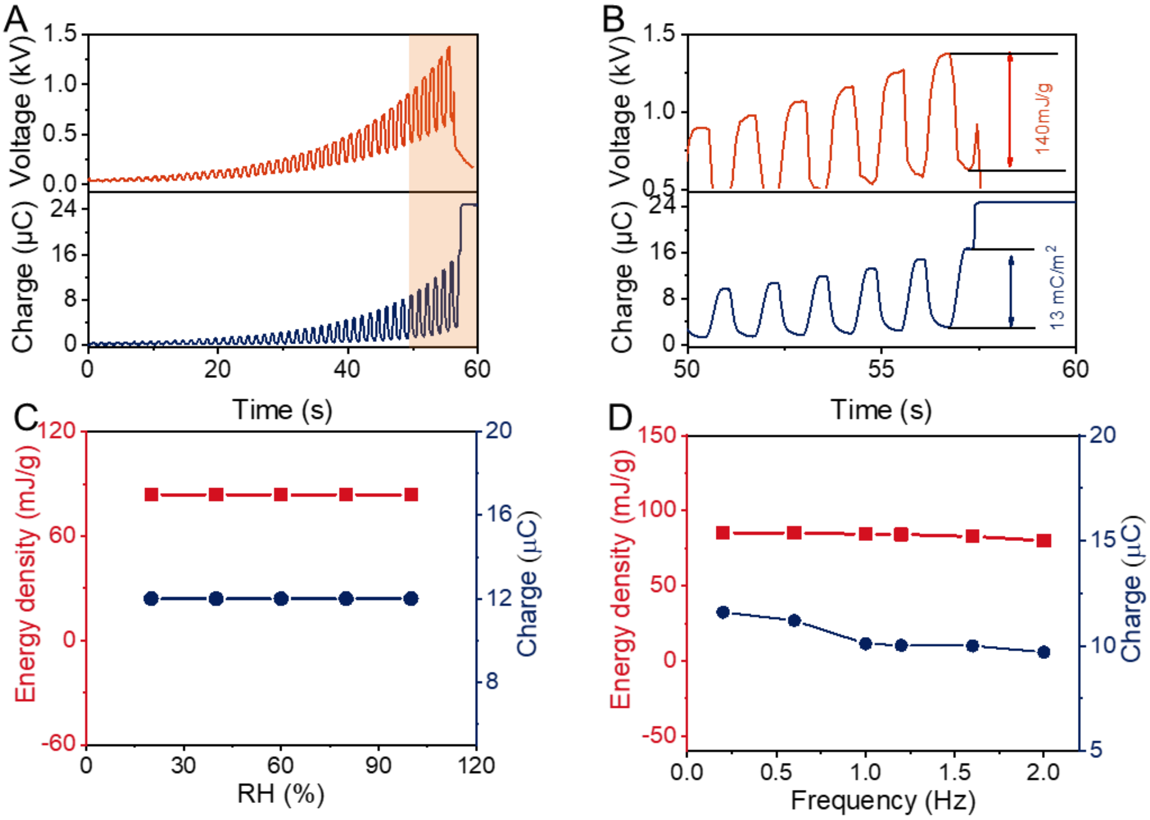

Figure S10 A Maximum output voltage and output charge of the DENG without the Zener diode. **B** Enlarged view of the shaded portion of yellow in **S10A**. Energy density and output charge of the DENG with **C** different humility and **D** different frequency.

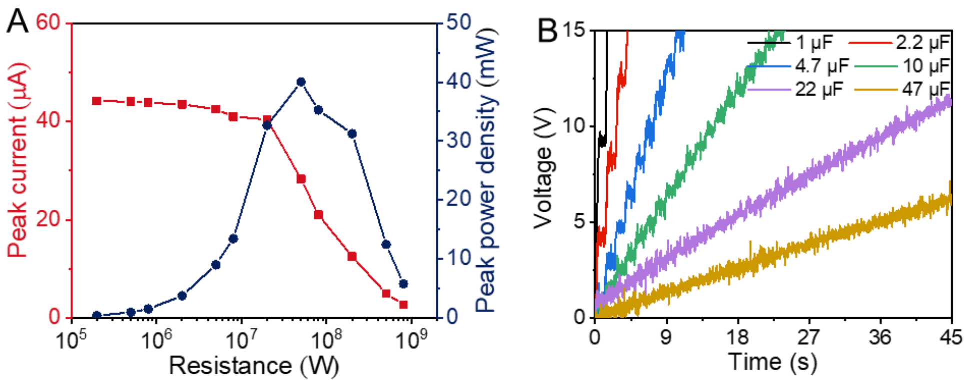

Figure S11 A Peak current and peak power with respect to the load resistance for the DENG with 10 cm^2. The operation frequency is 0.8 Hz. **B** Charging voltage curves for different capacitor by the DENG.

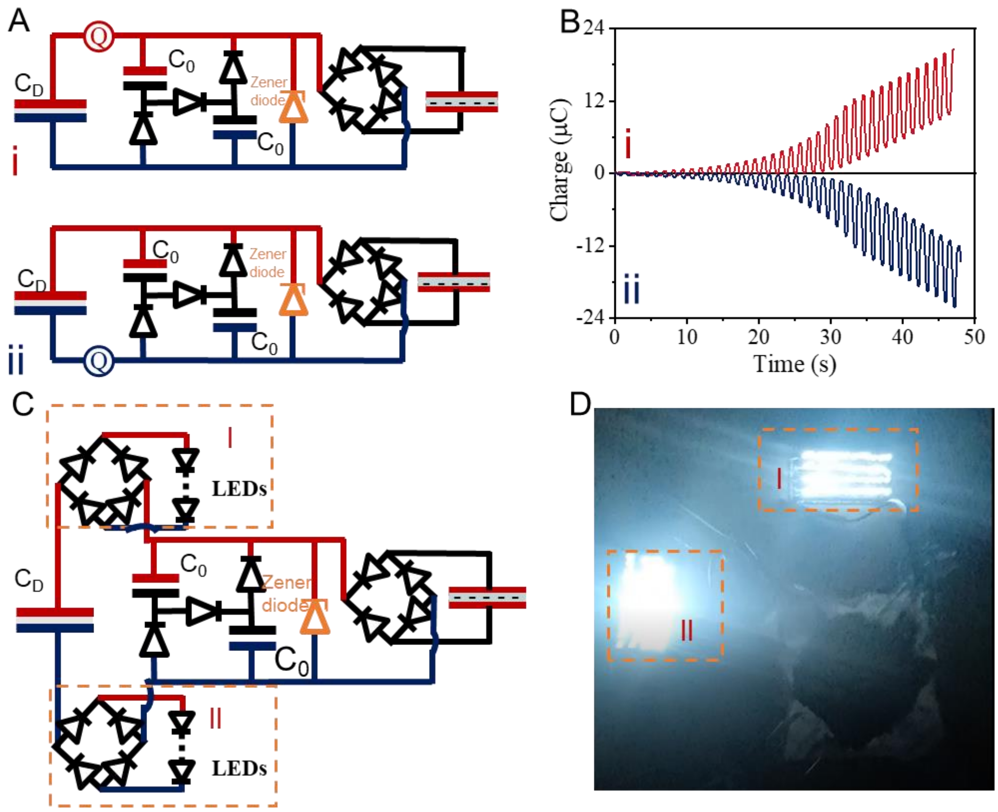

Figure S12 Measurement of output charges on both sides. A Schematic diagram of two steps for measuring the output charges on both side of the DENG. **B** Output charges of the DENG in the two steps. **C** Schematic diagram of lighting two arrays of 50 white LEDs, respectively on both sides. **D** Two arrays of 50 white LEDs were simultaneously lighted by the DENG.

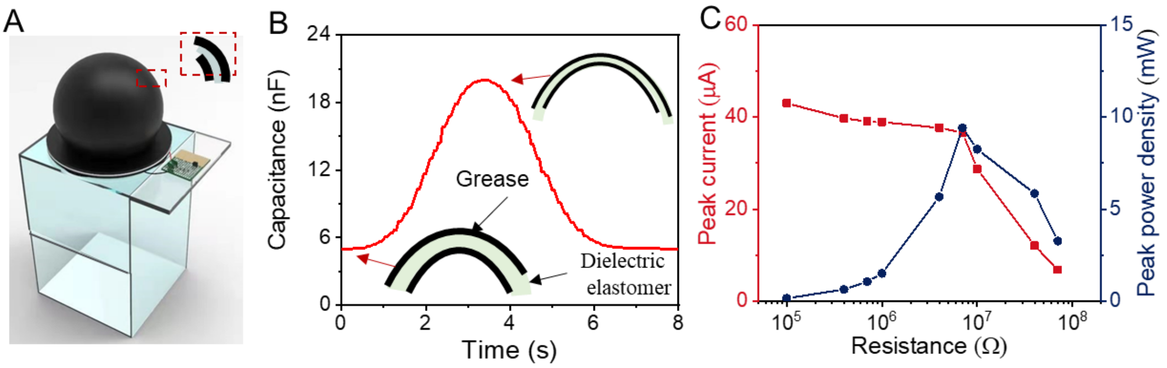

Figure S13. **A** Schematic scene of a single WEH vibrating in water wavers created by the standard wave tank equipment. **B** Capacitance change responses of the DEC under periodical deformations. Inset: schematic of inflation and deflation of the DEC. **C** Peak current and peak power of the integrated device with various loads.

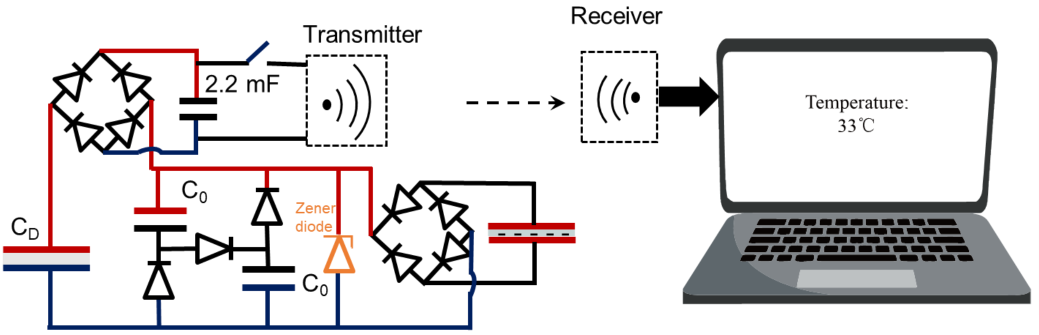

Fig S14 Schematic diagram of remote temperature reading system.

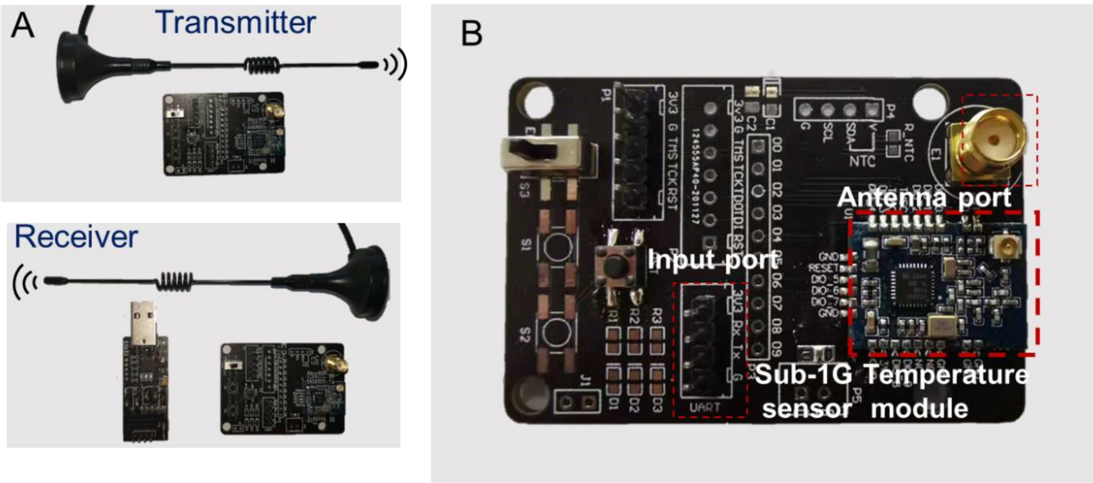

Figure S15 **Illustrations of remote temperature reading modules: a** transmitter (upper) and receiver (bottom); **b** port. Wireless module: CC1310. Temperature measurement module:433.
